# Non-negligible Water-permeance through Nanoporous Ion Exchange Medium

**DOI:** 10.1038/s41598-018-29695-x

**Published:** 2018-08-27

**Authors:** Jung A. Lee, Dokeun Lee, Sungmin Park, Hyomin Lee, Sung Jae Kim

**Affiliations:** 1 Department of Electrical and Computer Engineering, Seoul National University, Seoul, 08826 Republic of Korea; 2 Institute of Advanced Machines and Design, Seoul National University, Seoul, 08826 Republic of Korea; 3 Big Data Institute, Seoul National University, Seoul, 08826 Republic of Korea; 4 Inter-university Semiconductor Research Center, Seoul National University, Seoul, 08826 Republic of Korea; 5 Department of Chemical & Biological Engineering, Jeju National University, Jeju, 63243 Republic of Korea

## Abstract

While the water impermeable constraint has been conventionally adopted for analyzing the transport phenomena at the interface of electrolyte/nanoporous medium, non-negligible water-permeance through the medium results in significant effect on ion and particle transportation. In this work, a rigorous theoretical and experimental analysis of the water-permeance effect were conducted based on a fully-coupled analytical/numerical method and micro/nanofluidic experiments. The regime diagram with three distinctive types of concentration boundary layers (ion depletion, ion accumulation, and intermediate) near the ion exchange nanoporous medium was proposed depending on the medium’s permselectivity and the water-permeance represented by an absorbing parameter. Moreover, the critical absorbing parameters which divide the regimes were analytically obtained so that the bidirectional motion of particles were demonstrated only by altering the water-permeance without external stimuli. Conclusively, the presenting analysis of non-negligible water-permeance would be a substantial fundamental of transport phenomena at the interface of the ion exchange medium and electrolyte, especially useful for the tunable particle/ion manipulations in intermediate Peclet number environment.

## Introduction

Classically, a water-impermeable condition on the interface of a nanoporous medium and a bulk electrolyte has been generally chosen to analyze physicochemical transport phenomena because the internal volume of the nanoporous medium is usually smaller than the volume of outer electrolyte^1–3^. The condition is valid in the cases where the ionic flux induced by the water-permeance is negligible compared to the ionic flux due to the other external stimuli such as concentration difference^4–7^ and electric field^8–10^. For example, an ion concentration polarization phenomenon is triggered by electrically induced counter-ion flux across highly-charged nanoporous medium so that the theoretical model without considering the effect of water-permeance at the interface is enough to elucidate the transport phenomenon adequately^11–14^. However, in the case of reverse electrodialysis which is an energy harvesting platform utilizing a salt gradient across the nanoporous medium, diffusioosmotic water-permeance by diffusive flux could be a major transport mechanism^15–19^. In addition, it has been reported that a capillarity-driven water-permeance would naturally generate a permselective flux through the nanoporous medium, leading to the formation of ion depletion layer without external electrical power source^20,21^.

**Figure 1:**
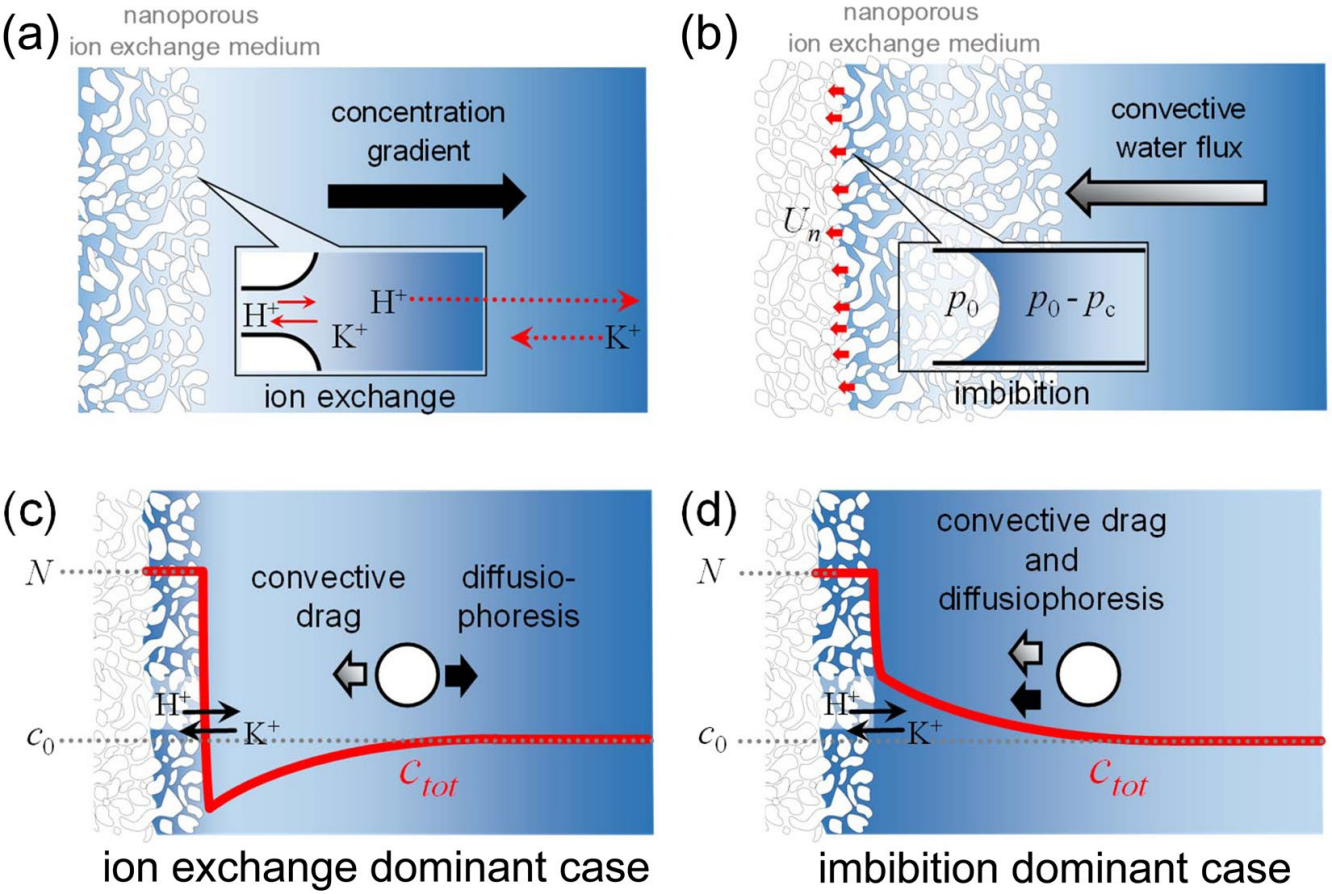
Two representative transport phenomena of nanoporous ion exchange medium; (**a**) concentration gradient induced by permselective ion exchange and (**b**) convective water flux toward the medium induced by imbibition. Concentration profiles near the water-absorbing ion exchange medium and the consequent drag force and the diffusiophoretic force exerted on a particle near the medium; (**c**) two forces acting on a charged particle are in the opposite direction with the concentration gradient toward the bulk (the ion exchange dominant case) and (**d**) both of the forces are the same direction with the concentration gradient toward the medium (the imbibition dominant case).

Similar to the capillarity-driven ion depletion layer, the salt gradient can be naturally formed by ion exchange process as well^22^. An internal proton would be exchanged with outer non-protonic cation through the Brownian motion of each ionic species. Classically, the total ion concentration outside the medium was treated as invariant because the exchange process was 1:1^23^. However, recent researches have proven that the difference of diffusion rate between the proton and dissolved cation results in the natural ion depletion layer^22^ as shown in Fig. 1(a). The concentration gradient inside the natural ion depletion layer provides an effective particle transporting mechanisms, known as diffusiophoresis^5–7,24^, so that one can observe the exclusion zone (EZ) of colloidal suspension, which is a particle-free zone expanding up to *O*(1) mm, adjacent to the ion exchange surface^25–33^. Both analysis and direct visualization of EZ formation provide abundant information (*e*.*g*. surface charge density, porosity, Donnan equilibrium quantities, *etc*) concerning the nanoporous material as well as the nanoscale ion exchange process. Hence, the natural ion depletion phenomenon induced by the ion exchange has been drawn significant attentions recently^22,25–31,33,34^. Despite the fact that the proton inside the medium is being dissociated by the absorbed water from the outer solution as shown in Fig. 1(b), the correlation between the capillarity-governed water-permeance through nanoscale pore and the natural ion depletion phenomenon (or EZ formation) still remains unknown.

Considering the effect of the water-permeance, the convective flow toward the ion exchange medium would affect the natural ion depletion and the EZ formation. When the water-permeating velocity is too small to perturb the natural ion depletion layer, the total ion concentration (*c*_*tot*_) nearby the ion exchange interface decreases as reported in previous literatures^22^ where the water-permeance was neglected. The direction of diffusiophoretic force on a colloidal particle is toward the bulk so that the concentration gradient-induced force would be competing with convective drag force arisen from the water-permeance as shown in Fig. 1(c). On the other hand, the concentration profile would be inverted if the water-permeating velocity becomes considerable as shown in Fig. 1(d). In such case, the forces on the colloidal particle have the same direction so that the EZ would be collapsed. These two limiting situations imply that the water-permeance would play a critical role in the natural ion depletion phenomenon and the EZ formation. Thus, in this work, a rigorous theoretical analysis was conducted to more precisely figure out the natural ion depletion phenomenon inhibited by the capillarity-governed water-permeance. Moreover, the theoretical findings were experimentally verified by the direct visualization of EZ formation.

## Theoretical Analysis

### Numerical modeling of concentration boundary layer near the ion exchange medium

The numerical simulations were conducted on two theoretical domains; Domain 1 is the interior of the nanoporous ion exchange medium, and Domain 2 is the bulk solution near the medium. The permselectivity of the medium was represented by the Donnan concentration (*N*) and the water-permeability was represented by the absorbing parameter (*S*). In the Domain 1, the water-permeance was described by the Darcy’s law as

1 $${U}_{n}=-\sqrt{\frac{\kappa {P}_{c}}{2\mu t}}\equiv -\sqrt{\frac{S}{t},}$$

where *U*_*n*_ is the water-permeating velocity into the medium, *κ* is the hydraulic permeability, *μ* is the viscosity of water, *t* is the time and *P*_*c*_ is the capillary pressure^35^. The Domain 2 was filled with electrolyte solution (*e*.*g*. KCl solution) with the bulk concentration of *c*_0_. In both domains, the transport of the three ionic species (K^+^, Cl^−^, and H^+^) was governed by the Poisson-Nernst-Planck equation as

2 $$\frac{\partial {c}_{i}}{\partial t}=-\,\frac{\partial }{\partial x}(-{D}_{i}\frac{\partial {c}_{i}}{\partial x}-\frac{{z}_{i}F{D}_{i}}{RT}{c}_{i}\frac{\partial \psi }{\partial x}+{c}_{i}{U}_{j}),$$

and

3 $$-\varepsilon {\nabla }^{2}\psi ={\rho }_{e}.$$

Here, *c*_*i*_ is the concentration of each ionic species *i*, *x* is the spatial coordinate, *D*_*i*_ is the corresponding ion’s diffusivity, *z*_*i*_ is the ionic valence, *F* is the Faraday constant, *R* is the gas constant, *T* is the absolute temperature, *ψ* is the electric potential, *U*_*j*_ is the flow velocity in Domain *j*, *ε* is the electrical permittivity of water, *ρ*_*e*_ is the volumetric charge density. The flow velocity in Domain 2 (*U*_*m*_) was determined by

4 $${U}_{m}={\phi }_{p}{U}_{n},$$

where *φ*_*p*_ was the porosity of the ion exchange medium. See Supplementary Note 1 for detailed physics.

### Simplification of the fully coupled model

When *N*/c_0_ $\gg$ 1, an ideal permselectivity condition (*i*.*e*. *j*_*Cl*_ = 0) enables the simplification of the fully-coupled model with the analytically obtained *c*_*tot*_ as

5 $${c}_{tot}(x,t)={c}_{0}erf(\frac{x}{\sqrt{4{D}_{K,eff}t}}+\sqrt{\frac{S}{{D}_{K,eff}}})+{c}_{H,S}[1-erf(\frac{x}{\sqrt{4{D}_{H,eff}t}}+\sqrt{\frac{S}{{D}_{H,eff}}})],$$

where *D*_*i*,*eff*_ is the effective diffusivity (*D*_*i*,*eff*_ = 2*D*_*i*_*D*_*Cl*_ /(*D*_*i*_ + *D*_*Cl*_)), and *c*_*H*,*S*_ is a constant defined as

6 $${c}_{H,S}\equiv {c}_{0}\frac{\sqrt{\frac{{D}_{K,eff}}{S}}{e}^{-\frac{S}{{D}_{K,eff}}}+2erf(\sqrt{\frac{S}{{D}_{K,eff}}})}{\sqrt{\frac{{D}_{H,eff}}{S}}{e}^{-\frac{S}{{D}_{H,eff}}}-2[1-erf(\sqrt{\frac{S}{{D}_{H,eff}}})]}.$$

**Figure 2:**
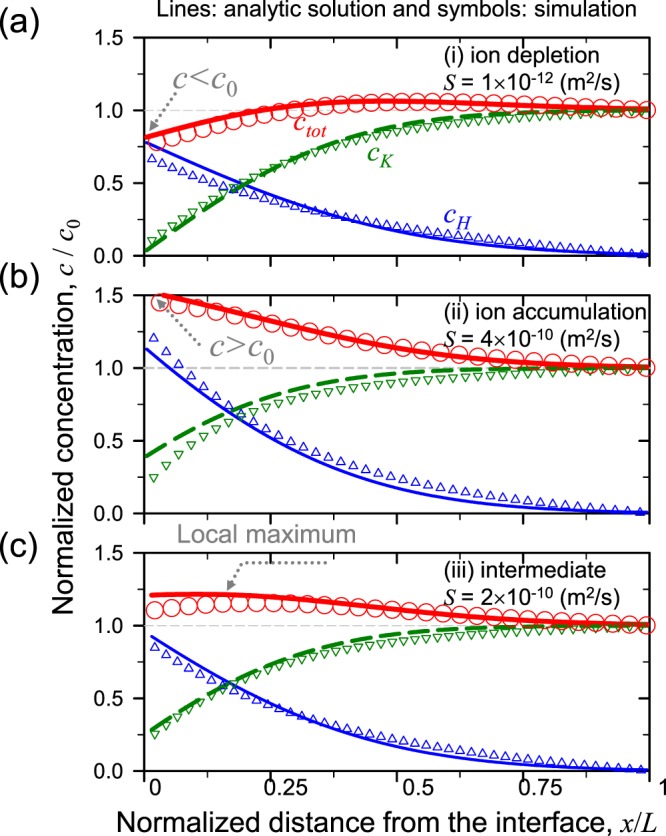
Representing concentration profiles and comparisons of analytic solutions to numerical ones for (**a**) *S* = 1 × 10^−12^ m^2^/s for the ion depletion, (**b**) *S* = 4×10^−10^ m^2^/s for the ion accumulation and (**c**) *S* = 2 × 10^−10^ m^2^/s for the intermediate.

The analytical solutions (symbols) and the numerical solutions (lines) of fully coupled model show a good agreement as shown in Fig. 2(a–c). See Supplementary Note 2 for detailed derivations.

## Results and Discussion

### Characterization of the concentration boundary layer with water-permeance

According to the results in Fig. 2(a–c), the characteristics of *c*_*tot*_ profile near the ion exchange medium is highly dependent on the water-permeability of the medium. The Fig. 2(a) shows the ion depletion when *S* is small as in the conventional water-impermeable condition. The thick red line (*c*_*tot*_) near the medium becomes smaller than *c*_0_ near the medium. Under this concentration gradient, the direction of diffusiophoresis is toward the bulk. On the contrary, when *S* is high as shown in Fig. 2(b), the *c*_*tot*_ near the medium is higher than *c*_0_ and monotonically decreases toward the bulk. With such a high water-permeance, ion exchange at the interface is suppressed because the diffusion of protons toward the bulk is hindered by the convective flow, leading to the ion accumulation near the interface. At this time, the direction of diffusiophoresis is toward the ion exchange interface. Figure 2(c) shows the intermediate situation where *c*_*tot*_ has a local maximum, while *c*_*tot*_ is higher than *c*_0_ near the medium. The position of the local maximum moves toward the bulk as a function of time. Therefore, charged particles near the medium moves to the bulk following the moving maximum so that the charged particles cannot be used as tracers to determine whether the total concentration near the medium is decreased or not, *i*.*e*. the particle behaves like the ion depletion, while actual ions are accumulated near the medium.

**Figure 3:**
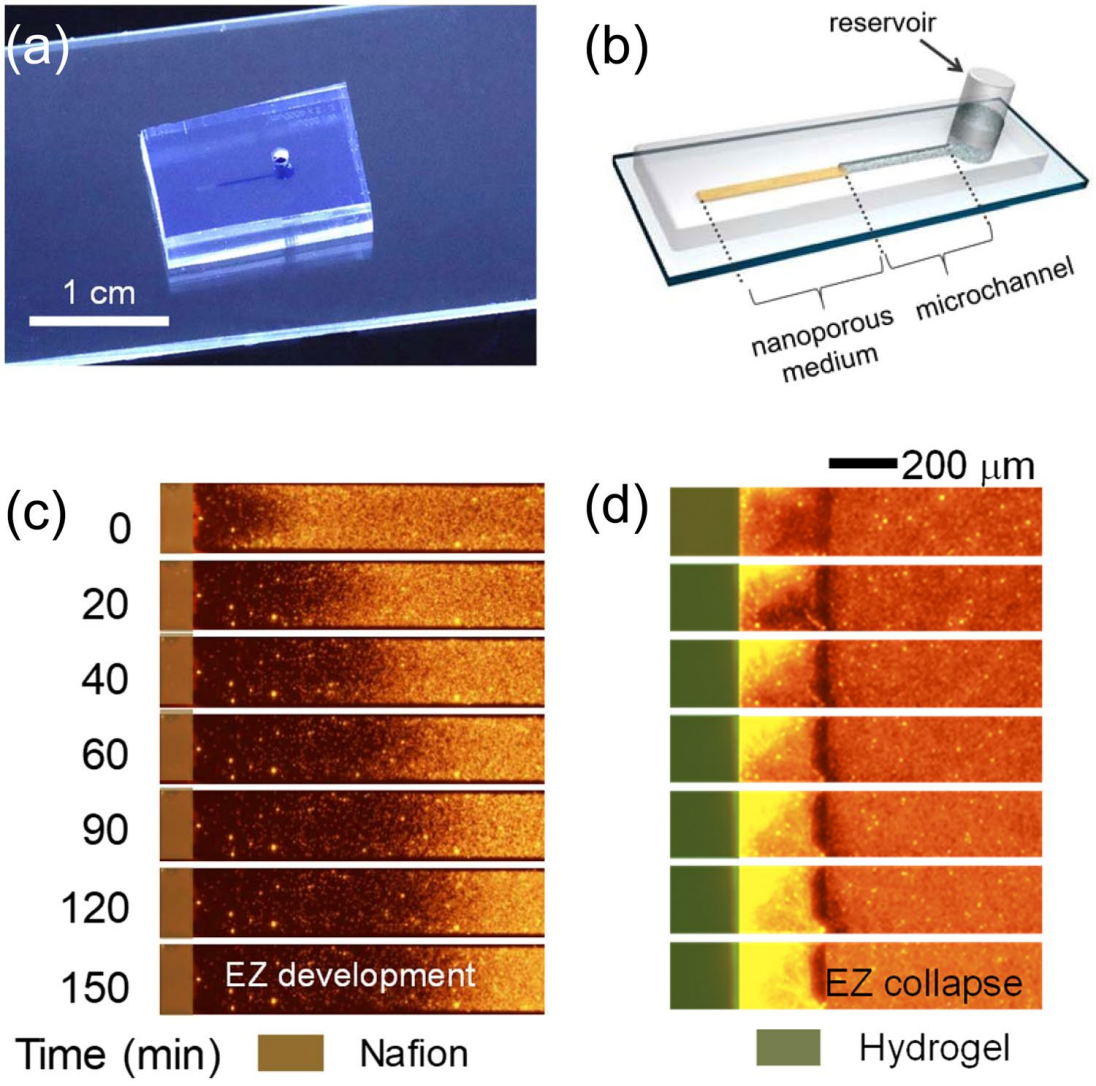
(**a**) The photo of an assembled microfluidic chip and (**b**) the schematics of the chip. Experimental demonstration of (**c**) the ion depletion using Nafion and (**d**) the ion accumulation using hydrogel.

These concentration profiles affected by the water-permeance were verified by direct visualization of EZ formation in pseudo 1-dimensional micro/nanofluidic chips shown in Fig. 3(a). The microfluidic chip had a reservoir at an end of the microchannel, and the other end was connected with a straight nanoporous medium as described in Fig. 3(b). As a building block of the microchannel, polydimethyl-siloxane (PDMS, Sylgard 184 silicone elastomer kit, Dow Corning, USA) was used. The ion exchange medium was either of Nafion (Sigma Aldrich, USA) or dry hydrogel which represented as materials having low or high water-permeability, respectively. See Supplementary Note 3 for detailed device fabrication and experimental setup.

Nafion which represented as the ion exchange medium of low water-permeability showed the developing EZ with the charged particles excluded to the bulk reservoir as shown in Fig. 3(c). On the other hand, dry hydrogel which simulated as the medium of high water-permeability showed the collapse of the EZ with an accumulation of charged particles as in Fig. 3(d). The fluorescent signal near the medium (left side of the microchannel) became brighter since the fluorescent particles were accumulated near the hydrogel medium. See Supplementary Video 1.

### The regime diagram

**Figure 4:**
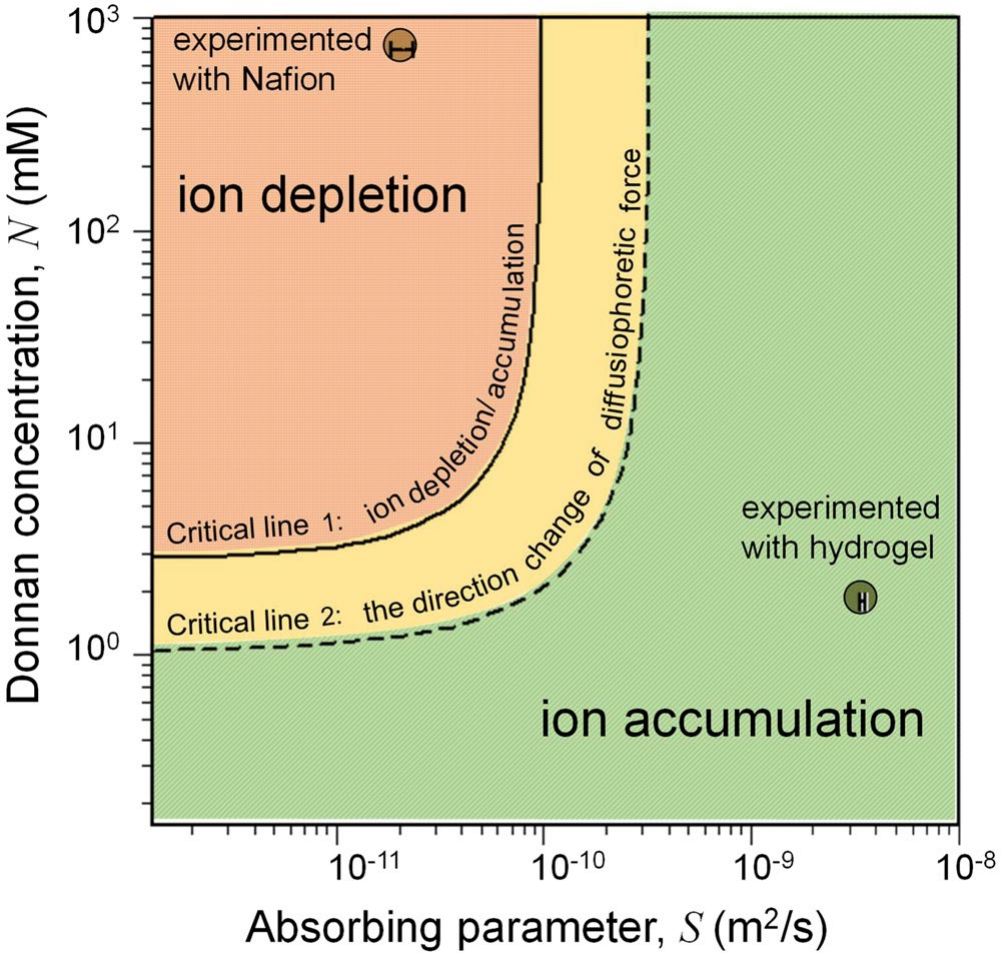
Regime diagram of three distinctive types of ion concentration profiles near the permselective ion exchange medium. The experimental results of Fig. 3(c) as ion depletion with *S* = 2.1×10^−11^ m^2^/s and *N* = 7.2 × 10^2^ mM and Fig. 3(d) as ion accumulation with *S* = 3.5 × 10^−9^ m^2^/s and *N* = 1.8 mM were examplified.

Based on this analysis, a regime diagram which also incorporates the correlation between the permselectivity (*N*) and the water-permeability (*S*) is proposed in Fig. 4. The regime diagram contains the three types of *c*_*tot*_ profiles (ion depletion, ion accumulation, and intermediate) with respect to *N* and *S*. The ion depletion occurs under high *N* and low *S* conditions while the ion accumulation occurs under the low *N* and high *S* conditions. According to the regime diagram, it is confirmed that the Nafion belongs to the ion depletion regime with the estimated *S* of 2.1 × 10^−11^ m^2^/s, and *N* of 7.2 × 10^2^ mM, and the dry hydrogel belongs to the ion accumulation regime with the estimated *S* of 3.5 × 10^−9^ m^2^/s and *N* of 1.8 mM. The *S* values were measured by tracking the particles near the reservoir where the diffusiophoretic force can be neglected, and the *N* values were estimated by measuring the conductance of a microchannel coated with the nanoporous medium. See Supplementary Notes 4 and 5 for detailed information.

The critical lines in the regime diagram are obtained using the *c*_*tot*_ and ∂*c*_*tot*_/∂*x* values at the interface (*x* = 0). The first critical line in Fig. 4 decides whether *c*_*tot*_ at the surface of ion exchange medium (*x* = 0) is higher than *c*_0_ or not, and the second critical line determines whether the local maximum of the concentration is formed or not. A notable observation on the critical lines is that the lines vertically divide the regimes when *N* > 10 mM (*i*.*e*. *N*/c_0_ $\gg$ 1). This indicates the regime is solely determined by the critical *S* values (*S*_*dep*_ and *S*_*acc*_) when *N* was high enough to assure the ideal permselectivity, and thus, the critical *S* values could be also obtained analytically as well as numerically. The analytically obtained values of *S*_*dep*_ and *S*_*acc*_ were 3.16 × 10^−11^ m^2^/s and 1.26 × 10^−10^ m^2^/s, respectively and the numerically obtained ones were 6.28 × 10^−11^ m^2^/s and 2.53 × 10^−10^ m^2^/s, respectively. The numerically obtained values were slightly greater than ones by analytical solution due to the ideal permselectivity assumption. However, they still convey a significant physical intuition since one can guarantee the direction of diffusiophoresis to the bulk if *S* was selected below 3.16 × 10^−11^ m^2^/s (analytically obtained *S*_*dep*_).

### Bidirectional particle motion

While aforementioned regime diagram based on 1-dimensional analysis brought the significant physical interpretation of a nanoporous medium, a practical 2- or 3-dimensional micro/nanofluidic configuration can alter water-permeance depending on the ratio of cross-sectional area of the medium to the microchannel. Once the medium was chosen (*i*.*e*. an *S* value was set), one can setup the two forces acting on a charged particle as in Fig. 1(c) or (d). The particle’s moving velocity (*U*_*p*_) is represented by the sum of two velocities as

7 $${U}_{p}={U}_{m}+{U}_{DP}\,{\rm{where}}\,{U}_{m}=-{\phi }_{p}\sqrt{\frac{S}{t}}\,{\rm{and}}\,{U}_{DP}=\frac{a}{\sqrt{t}}=\frac{{D}_{DP}}{{c}_{tot}}\frac{\partial {c}_{tot}}{\partial \eta }\frac{1}{\sqrt{t}}.$$

**Figure 5:**
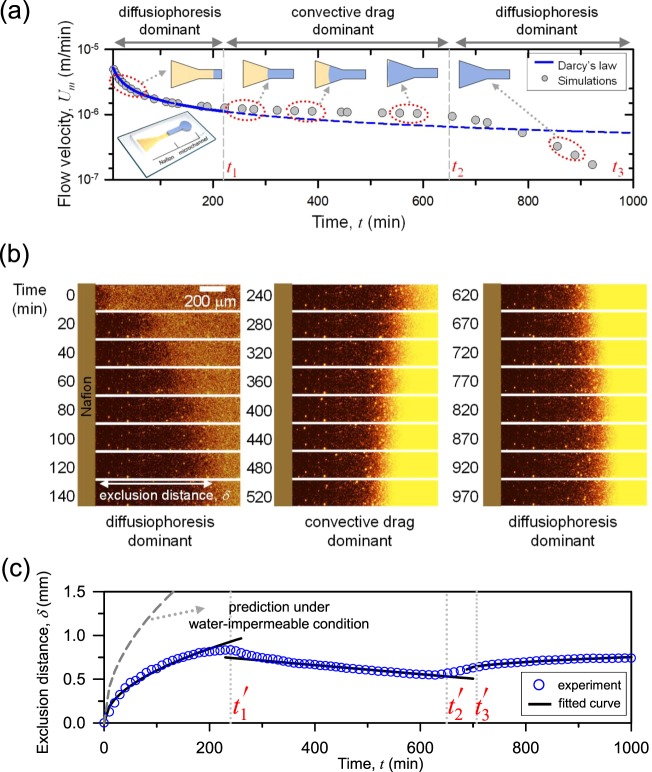
(**a**) Numerically estimated *U*_*m*_ in the microchannel generated from the imbibition through the funnel-shaped nanoporous ion exchange medium. (**b**) Time revolving images of charged particles’ motion using the funnel-shaped Nafion. (**c**) The plot of the particles’ exclusion distance as a function of time. Solid lines are regression curves proportional to *t*^*1/2*^, *t*, (*t* + *t*_3_^’^)^*1/2*^, respectively.

Here *U*_*m*_ is the flow velocity from Eqs (1 and 4) and *U*_*DP*_ is the diffusiophoretic velocity^22^ where *D*_*DP*_ is the diffusiophoresis constant depending on the zeta potential of a particle, viscosity of fluid, temperature, and diffusivity of ions^36^. Note that both of the velocities in Eq. (7) are inversely proportional to the square root of *t*, which indicates the direction of the particle’s motion should be unidirectional. Thus, if the particle moves to the bulk in the beginning, it keeps flowing to the bulk and *vice versa*. This was verified from the pseudo 1-dimensional experimental demonstration in Fig. 3(c) or (d). For example, the particle exclusion in Fig. 3(c) lasted for more than 16 hours until the ion exchange process ended after reaching the equilibrium. This was the limitation of pseudo 1-dimensional configuration where the direction of particle motion was uncontrollable. In this work, this unidirectional motion became a bidirectional motion through the higher-dimensional configuration to adjust the water permeable boundary condition. If the ion exchange medium was patterned as funnel shape as shown in the inset of Fig. 5(a), the flow velocity induced by the imbibition is expected to deviate from the *t*^−1/2^ dependency^37–40^. Figure 5(a) showed the simulated water-permeance based on the Richard’s equation^40^, and more information on the simulations is available in Supplementary Note 6. Until *t*_1_, water was absorbed through the straight pathway following the conventional Darcy’s law. After *t*_1_, a sudden increase of water influx induced by the diverging pathway led to the saturated imbibition velocity until *t*_2_^39^. Finally, the imbibition head reached the end of the medium at *t*_2_ and the water absorption dropped dramatically after *t*_2_. Finally, the medium was fully wetted at *t*_3_. Note that, as described in equation (7), the velocity of particle is the summation of imbibition velocity through microchannel and diffusiophoretic velocity. Also shown in equation (7), diffusiophoretic velocity is independent from geometrical dimension. However, change in geometric/channel dimensions affects the imbibition velocity (or the length of exclusion zone). It depends on the ratio of cross-section, *A*_*n*_*/A*_*m*_, as below.

8 $${A}_{m}{U}_{m}={A}_{n}{\phi }_{p}{U}_{n}$$

where *A*_*m*_: cross-sectional area of microchannel, *A*_*n*_: cross-sectional area of nanoporous medium, *φ*_*p*_: porosity of nanoporous medium and *U*_*n*_: velocity of the imbibition head in nanoporous medium. This is obtained from the flow continuity condition, and the bidirectional motion of charged particles was demonstrated by utilizing this relation. In that experiment, *A*_*n*_ was manipulated. Likewise, the dimension of a microchannel also has similar effects on the exclusion zone.

Based on this analysis, one can expect the direction shifts of charged particles’ motion and the experimental demonstration was provided in Fig. 5(b). See Supplementary Video 2. Figure 5(c) showed the exclusion distance (*δ*) of the charged particles extracted from Fig. 5(b). Until *t*_1_^’^, the system was in pseudo one-dimensional configuration with the water absorbed the straight pathway. The diffusiophoretic velocity was faster than the flow velocity in this region so that *δ* increased as a function of *t*^1/2^ until *t*_1_^’^. Between *t*_1_^’^ and *t*_2_^’^, flow velocity exceeded the diffusiophoretic velocity due to the expanding imbibition pathway. During the period, the particles moved backward to the ion exchange medium, and *δ* decreased proportional to time *t* due to the saturated flow velocity. Then the imbibition head reached the end of the funnel shaped medium and the flow velocity suddenly dropped at *t*_2_^’^. Finally, the medium was fully wetted and the particles were excluded again toward the bulk having *δ* proportional to (*t* + *t*_3_^’^)^1/2^ after *t*_3_^’^. This second exclusion lasted for more than 7 hours until the ion exchange reached the equilibrium. During the entire process, the motion of particles was governed by the shape of medium, while only one type of material was used (*i*.*e*. fixed *S*).

## Conclusions

As demonstrated, the nanoporous medium which can imbibe water using capillary force induces the convective flow toward the medium, and the permeable flow should affect the formation of the concentration boundary layer. The presenting analysis would provide (1) the regime diagram with three distinctive types of concentration profiles (ion depletion, ion accumulation, and intermediate) depending on the permselectivity and the water-permeance, (2) the critical *S* values (*S*_*dep*_, *S*_*acc*_) which divide the regimes, and (3) the practical utilities of the critical *S* values for bidirectional control of particle motion without external stimuli. Therefore, the new insight of critical role of water-permeance would be a seminal physics for interpreting transport phenomena at the interface of the ion exchange medium and electrolyte, especially useful for the tunable particle/ion manipulations in intermediate Peclet number environment.

## Electronic supplementary material

Spontaneous electrokinetics_video1

Spontaneous electrokinetics_video2

Supporting information
