# Supplementary material for: Non-negligible Water-permeance through Nanoporous Ion Exchange Medium
Source: Sci Rep. 2018 Aug 27;8:12842. doi: 10.1038/s41598-018-29695-x (PMC6110714; doi:10.1038/s41598-018-29695-x)
Supplement: Supplementary file 3 — Supporting information [file 41598_2018_29695_MOESM3_ESM.docx]

**Supplementary Note for**

Non-negligible Water-permeance through Nanoporous Ion Exchange Medium

Jung A Lee, Dokeun Lee, Sungmin Park, Hyomin Lee and Sung Jae Kim

**Note 1. Numerical simulation of concentration boundary layer**

The numerical simulation was conducted using a commercial software (COMSOL 4.4) on the domains consisting of the interior and the exterior of the ion exchange medium assuming the one-dimensional system with the spatial coordinate, *x*. The interior of the ion exchange medium denoted by Domain 1 in Fig. S1 (a) had the boundary at *x*=-*L_n_* (*t*) which moved to the left at the speed of imbibition velocity, *U_n_*.

$U_{n}=-\sqrt{\frac{S}{t}}$, (S1)

Domain 2 in Fig. S1 (a) represented the exterior of the ion exchange medium with the ion exchange surface at *x*=0 and the bulk at *x*=*L*. The imbibition through the interior of the ion exchange medium induced the flow velocity in both domains. The flow velocity in Domain 1 was the same as the imbibition velocity *U_n_*, and the flow velocity in Domain 2 denoted by *U_imb_* was

, (S2)

where *ϕ_p_* was the porosity of the ion exchange medium.

In both domains, the concentration of three ionic species (K^+^, H^+^, and Cl^-^) were described by the Nernst-Planck equations

$\frac{\partial c_{i}}{\partial t}=-\frac{\partial}{\partial x}\left(-D_{i}\frac{\partial c_{i}}{\partial x}-\frac{z_{i}FD_{i}}{RT}c_{i}\frac{\partial\psi}{\partial x}+c_{i}U_{j}\right)$, (S3)

where *c_i_* was the concentration of ionic species *i*, *t* was the time, *D_i_* was the corresponding ion’s diffusivity, *z_i_* was the ionic valence, *F* was the Faraday constant, *R* was the gas constant, *T* was the absolute temperature, *ψ* was the electric potential, and *U_j_* was the flow velocity in Domain *j*.

The electric potential, *ψ,* originated from the interaction between each ionic species and the charged groups at the medium surface was governed by the Poisson equation. The Poisson equation for Domain 1 was written as

$-\varepsilon\nabla^{2}\psi=F\left(c_{Na}-c_{Cl}+c_{H}-N\right)$, (S4)

where *ε* was the electrical permittivity of water and *N* was the Donnan concentration. Here, the Donnan concentration (*N*) represented the surface charge density of the nanoporous ion exchange medium. The Poisson equation for Domain 2 was

$-\varepsilon\nabla^{2}\psi=F\left(c_{Na}-c_{Cl}+c_{H}\right)$ (S5)

since the charge density of the bulk material was negligible.

At *x*=*L*, the electric potential was set to be the ground (*ψ*=0), and the boundary concentration of each ionic species was set to be its bulk concentration (*c_K_*, *c_Cl_* = *c*_0_ and *c_H_* = 0). At the moving boundary (*x*=-*L_n_* (*t*)), the flux conditions of K^+^ and Cl^-^ set to be the convective flux induced by the flow velocity as

$\left\{\begin{matrix} j_{K}=c_{K}U_{n} \\ j_{Cl}=c_{Cl}U_{n}. \end{matrix}\right.$ (S6)

On the other hand, the flux of H^+^ was

$j_{H}=\left(c_{H}-N\right)U_{n}$, (S7)

since additional protons were dissociated from the surface as the imbibition head moved. Also, by the conditions in Eq. (S6) and (S7), no electric current were generated at the moving imbibition head. Thus, zero-electric field condition was imposed at the moving boundary, *x* =-*L_n_* (*t*).

At the interface between electrolyte and the nanoporous medium, ions were exchanged satisfying zero electric current density condition of *j_H_+j_K_-j_Cl_-=* 0, where *j_i_* was the flux of ionic species *i*. When *N* was much higher than *c*_0_, anions were rejected from the interface due to the high negative Donnan potential formed in the nanopores (*i*.*e*. *j_Cl_=* 0). This led to the simplified flux condition of *j_H_+j_K_=* 0*,* which corresponded to the 1 to 1 cationic exchange.

The simulation results for *S* =10^-13^ m^2^/s, *c*_0_ =1 mM, *N* =100 mM,* ϕ_p_* =1, were shown in Fig. S1 (b). The imbibition head moved to the left as a function of square root of time *t*, while the length of the electrical double layer was fixed. Due to the permselective ion exchange, protons diffused to the bulk and the potassium ions were depleted.

Figure S1. (a) Description of domains for the numerical simulations. (b) Spatiotemporal concentration profiles.

**Note 2. Analytic solutions for the simplified model**

Simplification of the fully-coupled model is under the assumption of ideal permselectivity which can be represented by the cationic flux as,

$j_{K}+j_{H}=0$. (S8)

Also, the simplified model only covers the diffuse layer denoted in Fig. S1 (a) where the volume charge density is zero, and the concentration of chloride (*c_Cl_*) is expressed with the sum of *c_K_* and *c_H_* to satisfy the local neutrality as

$c_{K}+c_{H}=c_{Cl}$, (S9)

Using this, the concentration of chloride ions is divided into two terms as

$\left\{\begin{matrix} c_{Cl}^{H}=c_{H} \\ c_{Cl}^{K}=c_{K} \end{matrix}\right.$, (S10)

and the total ionic concentration is expressed as

$c_{tot}\equiv\frac{c_{K}+c_{H}+c_{Cl}}{2}=c_{K}+c_{H}$. (S11)

Using the Eq. (S8, S9) the two governing equations of Eq. (S3) and Eq. (S5) are combined and expressed as

$\frac{\partial c_{i}}{\partial t}=-\frac{\partial}{\partial x}\left(-D_{i,eff}\frac{\partial c_{i}}{\partial x}-c_{i}\sqrt{\frac{S}{t}}\right)$, (S12)

where the electromigration term is substituted using the effective diffusivity, *D_i,eff_* = *2D_i_D_Cl_*/(*D_i_+D_Cl_*). Then the analytic solution for *c_K_* satisfying the boundary conditions of

$\left\{\begin{matrix} \left.c_{K}\right|_{x=\infty}=c_{0} \\ \left.c_{K}\right|_{x=0}\approx0 when \frac{S}{D_{K,eff}}\ll1 \end{matrix}\right.$ (S13)

is obtained using the method of combined variables (*η*=*x*/*t*^1/2^) as [1]

$c_{K}\left(x,t\right)=c_{0}erf\left(\frac{x}{\sqrt{4D_{K,eff}t}}+\sqrt{\frac{S}{D_{K,eff}}}\right)$. (S14)

Using this, the analytic solution of *c_H_* satisfying the boundary conditions of

$\left\{\begin{matrix} \left.c_{H}\right|_{x=\infty}=0 \\ -D_{H,eff}\left.\frac{\partial c_{H}}{\partial x}\right|_{x=0}-\left.c_{H}\right|_{x=0}\sqrt{\frac{S}{t}}=D_{K,eff}\left.\frac{\partial c_{K}}{\partial x}\right|_{x=0}+\left.c_{K}\right|_{x=0}\sqrt{\frac{S}{t}} \end{matrix}\right.$ (S15)

is obtained as

$c_{H}(x,t)=c_{H,S}\left[1-erf\left(\frac{x}{\sqrt{4D_{H,eff}t}}+\sqrt{\frac{S}{D_{H,eff}}}\right)\right]$, (S16)

with $c_{H,S}=c_{0}\frac{\sqrt{\frac{D_{K,eff}}{S}}e^{-\frac{S}{D_{K,eff}}}+2erf\left(\sqrt{\frac{S}{D_{K,eff}}}\right)}{\sqrt{\frac{D_{H,eff}}{S}}e^{-\frac{S}{D_{H,eff}}}-2\left[1-erf\left(\sqrt{\frac{S}{D_{H,eff}}}\right)\right]}$. (S17)

The first critical line in the regime diagram in the main text (Fig. 3) decides whether *c_tot_* at the surface of ion exchange medium (*x=*0) is higher than *c*_0_ or not. Therefore, the first critical *S* value (*S_dep_*) is obtained from the condition of

$c_{tot}(0,t)=c_{0}.$ (S18)

The second critical line determines whether the local maximum of the concentration is formed or not. When the local maximum exists, the concentration gradient at *x=*0 should be positive. Therefore, the condition for the second critical *S* value (*S_acc_*) is

$\left.\frac{\partial c_{tot}}{\partial x}\right|_{x=0}=0$. (S19)

In order to obtain critical *S* values, the analytical solution of *c_tot_*|*_x_*_=0_ (Eq. (S14)|*_x_*_=0_ + Eq. (S16)|*_x_*_=0_) and ∂*c_tot_*/∂*x*|*_x_*_=0_ were plotted as shown in Fig. S2. From the intersection of *c_tot_*|*_x_*_=0_ and *c_tot_*/*c*_0_=1 ∂*c_tot_*/∂*x*|*_x_*_=0_ and *y*=0, the quantitative values of *S_dep_* and *S_acc_* were obtained as 3.16x10^-11^ m^2^/s and 1.26x10^-10^ m^2^/s, respectively.

Figure S2 The plot of *c_tot_*|*_x_*_=0_ and ∂*c_tot_*/∂*x*|*_x_*_=0_ using Eq. (S14,S16). From the intersection of *c_tot_*|*_x_*_=0_ and *c_tot_*/*c*_0_=1, ∂*c_tot_*/∂*x*|*_x_*_=0_ and *y*=0, the quantitative values of *S_dep_* and *S_acc_* were obtained as 3.16×10^-11^ m^2^/s and 1.26×10^-10^ m^2^/s, respectively. (b) Representing concentration profiles and comparisons of analytic solutions to numerical ones for each regime (*S*=10^-12^ m^2^/s for the ion depletion regime, *S*=10^-9.4^ m^2^/s for the ion accumulation regime and *S*=10^-9.7^ m^2^/s for the intermediate regime).

**Note 3. Device fabrication and experimental setup**

The microfluidic chips was fabricated as shown in Fig. S3. As a building block of the microchannel, polydimethyl-siloxane (PDMS, Sylgard 184 silicone elastomer kit, Dow Corning, USA) was used. The base and curing agents of PDMS were mixed in a ratio of 10:1, and degassed in a vacuum chamber for one hour. The mixed solution was poured onto a silicon wafer which was patterned with microchannels and was cured in an oven for four hours at 75 °C.

The device using Nafion (Sigma Aldrich, USA) as a nanoporous medium was made of a PDMS block and a glass slide as shown in Fig. S3 (a). The Nafion was patterned on the glass slide following a previously reported surface patterning method [2] with the dimension of 200 μm (width) × 0.5 μm (depth) × 1.5 mm (length). On the Nafion-patterned glass slide, the prepared PDMS block was irreversibly bonded to a designated position using plasma bonder (CuteMP, Femto Science, Korea). The microchannel has the dimension of 200 μm (width) × 15 μm (depth) × 5 mm (length) and filled with a mixture of 1mM KCl solution (Sigma Aldrich, USA) with the negatively charged fluorescent polystyrene particles (diameter = 200 nm, Invitrogen, USA).

The device using hydrogel as a nanoporous medium was made of two layers of PDMS blocks and a glass slide as shown in Fig. S3 (b). The microchannel in the bottom PDMS block was filled with a UV curable hydrogel precursor, a mixture of 2-hydroxyethyl methacrylate (Sigma Aldrich, USA), acrylic acid (Sigma Aldrich, USA), ethylene glycol dimethacrylate (Sigma Aldrich, USA)), and 2,2-dimethoxy-2-phenylacetophenone (Sigma Aldrich, USA) at the weight ratio of 32.51: 18.041: 1.25 [3]. The other microchannel in the top PDMS block was irreversibly bonded onto the bottom microchannel in a T-shape as shown in Fig. S2 (b), and filled with a mixture of KCl solution with microparticles as in the device using Nafion. The dimension of the bottom microchannel (dimension of hydrogel) is 400 μm (width) × 50 μm (depth) × 16 mm (length), and the top microchannel has the dimension of 200 μm (width) × 15 μm (depth) × 5 mm (length).

In both devices, the convective flow induced by the imbibition through the nanoporous medium follows the 1-dimensional Darcy’s law since the cross-sectional area of the imbibition heads was fixed. Even in the case of the T-shaped hydrogel device having two imbibition heads, the convective flow in the bulk microchannel follows the 1-dimensional Darcy’s law since each of the imbibition heads follows the law. Therefore, the pseudo 1-dimensional assumption was valid for both configurations.

Figure S3. Snapshots of (a) Nafion chip and (b) hydrogel chip.

**Note 4. Measurement of the Donnan concentration**

The Donnan concentrations of Nafion and hydrogel were estimated by measuring the ionic conductance of the microfluidic device incorporated with the nanoporous medium. As shown in Fig. S4(a), the device was comprised of a straight microchannel on top of the nanoporous medium (Nafion or hydrogel) patterned parallel to the microchannel.

To estimate Donnan concentration of Nafion and hydrogel properly, two slightly different devices were fabricated. For the Nafion chip, the Nafion was patterned on a slide glass with the dimension of 200μm (width) × 0.5μm (depth) × 4.5mm (length) and the top microchannel had the dimension of 200μm (width) × 15μm (depth) × 4.5mm (length). For the hydrogel chip, the liquid hydrogel was filled in the bottom microchannel whose dimension is 200μm (width) × 50μm (depth) × 20mm (length) and cured, and the top microchannel had the dimension of 250μm (width) × 50μm (depth) × 20mm (length). Both of the devices were filled with KCl solution and rested for about 1 hours until the nanoporous medium was fully wetted. Using these devices, conductance of the microchips were measured by changing the concentration of KCl solution as shown in the Fig. S4(b-c).

The conductance of device (*G_tot_*) is represented as

$G_{tot}=G_{m}+G_{n}$ (S20)

with $G_{m}=\frac{FA_{m}c_{0}(\mu_{+}+\mu_{-})}{2L_{m}},$ (S21)

and $G_{n}=\frac{FA_{n}}{2L_{n}}\left[\mu_{+}\left(\sqrt{4c_{0}^{2}+N^{2}}+N\right)+\mu_{-}\left(\sqrt{4c_{0}^{2}+N^{2}}-N\right)\right],$ (S22)

where *G_m_* is the conductance of top microchannel filled with electrolyte solution, and *G_n_* is that of the nanoporous medium, *A_m_* is the cross-sectional area of microchannel, *c*_0_ is the concentration of electrolyte solution, *μ*^+^ is the electrophoretic mobility of cation, *μ*^-^ is the electrophoretic mobility of anion, *L_m_* is the length of the top microchannel, *A_n_* is the cross-sectional area of nanoporous medium, *L_n_* is the length of the nanoporous medium, and *N* is the Donnan concentration [4]. Using the devices of *A_m_*> *A_n_*, the total conductance is mainly determined by *G_m_* with its value linearly proportional to *c_0_*. However, when *c_0_*<<*N*, the effect of *N* predominates the effect of *c*_0_, and the total conductance is determined by *G_n_* . In Fig. S3 (b-c), the plateau below the threshold concentration occurs when *G_n_* is dominant while the linearly increasing shape above the threshold concentration occurs when *G_m_* is dominant. The Donnan concentration was estimated through fitting the experiment points using the Eq. (S8-S10), and the estimated Donnan concentration of Nafion was 7.2×10^2^ mM and hydrogel was 1.8mM.

Figure S4. Measurement of the Donnan concentration. (a) Schematics of the devices incorporated with nanoporous medium. (b) The conductance of the Nafion device, and (c) hydrogel device.

**Note 5. Measurement of the imbibition-induced flow velocity**

The flow velocity induced by imbibition through a nanoporous medium was measured by tracking the motion of the fluorescent microparticles near the reservoir of the microchannel where the diffusiophoretic force can be neglected. The images of the particle’s motion were captured every 10 seconds using a commercial computer software (CellSens, Olympus, Japan) and an inverted fluorescent microscope (IX 53, Olympus, Japan). Every 10 seconds, the average velocity of particles were estimated, and the average velocity values were plotted in Fig. S5. Consequently, through the regression line derived from the Darcy’s law, the absorbing parameter (*S*) of Nafion and hydrogel is 2.1×10^-11^ m^2^/s (standard deviation: 2.7×10^-12^ m^2^/s) and 3.5×10^-9^ m^2^/s (standard deviation: 1.1×10^-10^ m^2^/s), respectively.

Figure S5. Flow velocity induced by the imbibition through nanoporous medium of (a) Nafion and (b) hydrogel.

**Note 6. Numerical simulation of the imbibition through an expanding pathway**

Imbibition of liquid in a porous medium is described by the Darcy’s law as

$\mathbf{q}=-\frac{k}{\mu}\nabla P,$ (S23)

where **q** is the flux of liquid normalized by its specific density, *k* is the permeability, *μ* is the viscosity of the liquid, and ∇*P* is the pressure gradient generated from the capillary pressure (*P_c_*) at the imbibition head. Combining the Darcy’s law with the 2-dimensional continuity equation of an incompressible fluid leads to

$\frac{\partial\theta}{\partial t}=\nabla\cdot\left(\frac{k}{\mu}\frac{\partial P}{\partial\theta}\frac{\partial\theta}{\partial x}\mathbf{e}_{x}+\frac{k}{\mu}\frac{\partial P}{\partial\theta}\frac{\partial\theta}{\partial y}\mathbf{e}_{y}\right)$. (S24)

Here, *θ* is the volumetric content of liquid defined as the volume of liquid divided by the void volume of the porous medium. For example, the *θ* value of dry part of the medium is 0 while the *θ* value of fully-wetted part is 1. The continuity equation in Eq. (S24) is analogous to the Fick’s laws of diffusion, so by using an equivalent diffusivity, *D* ≡ *k*∂*P*/*μ*∂*θ*, Eq. (S24) is rewritten as

$\frac{\partial\theta}{\partial t}=\nabla\cdot\left(D\frac{\partial\theta}{\partial x}\mathbf{e}_{x}+D\frac{\partial\theta}{\partial y}\mathbf{e}_{y}\right)=D\nabla^{2}\theta$. (S25)

This equation is known as the Richard’s equation [5]. Unlike the convectional Darcy’s law, the permeability (*k*) and capillary pressure (*P_c_*) are not constant but highly dependent on *θ*. Therefore, the equivalent diffusivity (*D*) is a function of *θ* as

$D=D_{0}\theta^{n},$ (S26)

where the exponent *n* is related to the pore size distribution [5].

Using Eq. (S25-S26), the imbibition through a nanoporous medium patterned in an expanding shape as shown in Fig. S6 was numerically simulated using COMSOL commercial software (COMSOL 4.3a) and the flow velocity (*U*) was estimated by integrating the influx of water through the bottom wall. The water absorption through the patterned medium was visualized in Fig. S6, with the simulation conditions of *n* =6, *D*_0_ =1.5×10^-10^ m^2^/s.

Figure S6. Numerical simulation results of imbibition through an expanding nanoporous medium.

[1] R. B. Bird, W. E. Stewart, and E. N. Lightfoot, *Transport phenomena* (J. Wiley, New York, 2002), 2nd, Wiley international edn.

[2] J. H. Lee, Y. A. Song, and J. Y. Han, Lab Chip **8**, 596 (2008).

[3] S. Park, Y. Jung, S. Y. Son, I. Cho, Y. Cho, H. Lee, H. Y. Kim, and S. J. Kim, Nat Commun **7** (2016).

[4] J. Kim, I. Cho, H. Lee, and S. J. Kim, Sci Rep-Uk **7** (2017).

[5] A. Perez-Cruz, I. Stiharu, and A. Dominguez-Gonzalez, Microfluidics and Nanofluidics **21** (2017).
